# Patient Safety in Ulcer Management: Healthcare Professionals’ Experiences of Systemic Challenges Across Primary and Secondary Healthcare Settings

**DOI:** 10.3390/healthcare14131925

**Published:** 2026-07-01

**Authors:** Marcus Rosenburg, Ingrid Larsson, Petra Svedberg, Anna Gyberg

**Affiliations:** School of Health and Welfare, Halmstad University, P.O. Box 823, 30118 Halmstad, Sweden; ingrid.larsson@hh.se (I.L.); petra.svedberg@hh.se (P.S.); anna.gyberg@hh.se (A.G.)

**Keywords:** healthcare professionals, patient safety, primary health care, qualitative research, secondary health care, ulcer management

## Abstract

**Highlights:**

The diversity and scope of ulcer management make it a demanding clinical field. Patient safety in ulcer management is complex, as delays in diagnosis increase the risk of incorrect treatment and may ultimately lead to prolonged healing and adverse events. Healthcare professionals’ experiences of patient safety are an important starting point towards ensuring safe, high-quality care for patients with ulcers worldwide.

**What are the main findings?**

- More could be done to organise and structure ulcer management to improve patient safety.
- Healthcare professionals involved in ulcer management were aware of patient safety risks and actively strived to minimise harm.

**What are the implications of the main findings?**

- In contexts characterised by time constraints and a sustained commitment among healthcare professionals to deliver high-quality care, investment in well-structured competence support for ulcer management may facilitate more timely diagnosis and more effective utilisation of specialist consultation.
- Over time, investment in organisational support and structures could contribute to the optimisation of clinical pathways, clearer delineation of levels of care, and improved role definition.

**Abstract:**

**Background/Objectives**: Ulcers affect people of all ages worldwide, and hard-to-heal ulcers require extensive management. Patients with hard-to-heal ulcers often receive care across multiple healthcare settings, which may increase risks to patient safety. There is limited knowledge of how healthcare professionals maintain patient safety when patients with hard-to-heal ulcers transition across healthcare settings. The aim of this study was to explore healthcare professionals’ experiences of patient safety in ulcer management in primary and secondary healthcare settings. **Methods**: A qualitative research design was adopted. Fifteen semi-structured interviews were conducted, transcribed, and analysed, using qualitative content analysis. The healthcare professionals represented various professional roles and worked in either primary or secondary care. **Results**: The study resulted in one overarching theme and three categories. Given the complexity and demands of ulcer management, healthcare professionals needed to ensure safe care. In an organisation with unclear structures, professionals felt compelled to safeguard and develop their expertise themselves. Limited formal training in ulcer management, along with a reliance on a few more experienced colleagues, contributed to a sense of inadequacy among healthcare professionals. In ulcer management, professionals acted in the patient’s best interests, helping address gaps in patient safety. **Conclusions**: The findings of the present study illustrated how healthcare professionals are largely left alone in ulcer management. Challenges due to organisational structures posed patient safety risks, which were, to a large extent, mitigated by the professionals’ dedication to patient care. This study highlights the need for stronger structural support to complement individual competence in promoting safe and effective ulcer management. Patient safety should not depend solely on individual professionals, but on structured, organisation-level guidelines.

## 1. Introduction

Ulcers affect people worldwide, and the prevalence of hard-to-heal ulcers is estimated to be 1% [1]. The human burden associated with hard-to-heal ulcers is reflected in various ways, including pain, chronic morbidity, deterioration of mobility [2], psychological distress, social isolation, anxiety, prolonged hospitalisation, and, in severe cases, mortality [3]. This burden has been shown to be alleviated through timely diagnosis [4], well-developed treatment strategies [5], and coordinated care [6]. However, in the context of fragmented care, patients with hard-to-heal ulcers often transit between different levels of healthcare, across home care, primary care, emergency departments, and inpatient settings. This discontinuity increases the risk of delayed healing and the development of additional adverse events and suffering [4,6]. Although there is a substantial body of knowledge regarding the prevention of ulcer development and recurrence, and strategies to promote healing, the burden associated with hard-to-heal ulcers and related adverse events remains significant. In this study, we seek to address how healthcare professionals ensure patient safety in the daily clinical ulcer management in primary and secondary healthcare settings.

‘Hard-to-heal ulcer’ is a frequently used term to describe a complexity in healing for various types of aetiology. The term covers various types of ulcers, including diabetic, venous, arterial, and pressure ulcers [7,8,9]. The most common hard-to-heal ulcer on the lower leg has venous aetiology in about 70% of cases. A recent study highlights that 8% of over 500,000 US nursing home residents had a pressure ulcer, graded 1 or higher [10]. The prevalence of diabetic foot ulcers worldwide has been reported at 6.3%, with the highest prevalence in North America, at 13% [11]. Approximately 4% of lower leg ulcers are arterial ulcers, which result from arterial occlusion and lead to reduced tissue perfusion and hypoxia [12]. The complexity of the underlying causes means that healing is often prolonged, with a documented median healing time in Sweden of 22 weeks for hard-to-heal ulcers [13]. Several types of ulcers, including those caused by pressure, diabetes, venous insufficiency, and arterial insufficiency, require a comprehensive treatment approach to promote healing [14].

In this study, we use the concept of ulcer management as an umbrella term for all elements involved in the assessment, treatment, follow-up, and coordination of care for patients with ulcers, with particular relevance to hard-to-heal ulcers that often require prolonged and coordinated management. Ulcer management depends on an accurate diagnosis, which requires an investigative procedure that includes data on the type of ulcer, overall health status, medications, and nutritional status [15]. Ulcer management includes ulcer cleansing, pain relief, pressure relief, medical podiatry, ulcer-specific dressing, and compression [16,17]. The patient’s capacity for self-care must be assessed, and based on the patient’s lifestyle, smoking cessation, reduced alcohol consumption, dietary changes, and increased or modified physical activity may be recommended [16,17,18]. Furthermore, ulcer management is collaborative, involving nurses, physicians, nursing assistants, and other healthcare professionals [19,20]. Accordingly, ulcer management can be understood as an extensive process that requires a strong knowledge base amongst professionals.

Despite advances in care, many patients still experience harm, a large proportion of which could have been avoided [21,22,23]. Common adverse events related to ulcer management, which are defined as avoidable injuries or suffering resulting from medical management [24], include the development of pressure ulcers, ulcer infections, and complications caused by delayed diagnosis or inadequate treatment [4,25]. Factors such as time, continuity of care, communication, and professional competence have been associated with an increased risk of preventable patient safety incidents [26]. Thus, patient safety in ulcer management encompasses both timely and appropriate management of existing ulcers to promote healing and prevent adverse events, as well as measures to prevent the development of new ulcers. However, although nurses often report familiarity with ulcer pathophysiology and aetiology, their perceived ability to perform ulcer assessments may be lower, suggesting that knowledge does not always translate into practice [7,27]. The importance of a timely diagnosis is illustrated by Ahmajärvi et al. [4,28], who highlight that delays in ulcer diagnosis are often driven by organisational factors, particularly in primary care, rather than patient behavior, and that prolonged or delayed diagnosis is associated with extended healing times and more severe outcomes [4,28]. Prompt identification of ulcers is therefore critical to ensure that appropriate aetiological treatment can be initiated and patient safety maintained.

Several areas have been identified as potential threats to patient safety, including high workload, low staffing, insufficient communication, and ineffective leadership [29,30]. Such circumstances may also adversely impact the development of a trusting relationship between healthcare professionals and patients, and their relatives [31,32,33], which is critical for ensuring patient safety [31,34]. However, previous research indicates that patients undergoing ulcer management often experience low trust in professionals [35]. In a literature review, Villar et al. (2020) found that patients attributed some of the causes of unsafe care to inadequate communication, both with patients and among healthcare professionals, and to a lack of respect or attentiveness from professionals, along with fragmented care [36]. Furthermore, patients perceived that no one maintained a comprehensive or coordinated overview of their care trajectory [37].

As outlined in existing research and suggested by Hollnagel et al. [38], patient safety is not a linear process; rather, it is characterised by complexity and variability, as situations unfold under continuously changing conditions and involve multiple interacting actors. The current study involved both primary and secondary care settings, with differing organisational mandates and multiple actors involved in ulcer management, including healthcare professionals in various roles, patients, and their relatives. Patient safety is conceptualised as a continuous, adaptive, and flexible approach that aims to protect patients from harm. This implies that patient safety is enacted and negotiated by all actors involved and evolves in response to how situations unfold in everyday practice [38]. From Hollnagel et al.’s [38] so-called safety-II perspective, patient safety can be understood as contextually situated rather than something that can be fully predefined or anticipated. Accordingly, patient safety work is viewed as comprising ongoing negotiations with patient safety actions, including non-actions, across a range of situations. Drawing on Sahlsten et al. [39], participation, here understood within the context of patient safety work, requires dynamic interactions grounded in “a foundation of interpersonal procedure, therapeutic approach, and a focus on resources and opportunities for influence” (p. 635) [39]. This perspective on patient safety enables an exploration of how healthcare professionals experience, maintain, and adapt patient safety in ulcer management in complex, real-world situations.

Overall, previous research demonstrates that hard-to-heal ulcers require coordinated multidisciplinary input across care settings, as diverse aetiologies necessitate complex, individualised management strategies tailored to both the specific ulcer type and the patient’s overall health status [14,19,20]. However, despite this complexity, existing research has largely addressed clinical outcomes and treatment strategies, with less attention to how patient safety is enacted and maintained in everyday practice across organisational boundaries. The prolonged management of hard-to-heal ulcers and the involvement of various levels of healthcare increase the risk of care fragmentation, which can lead to adverse events and patient suffering. Nevertheless, there remains a limited understanding of how these risks are experienced, negotiated, and managed by professionals within the complexities of real-world practice. Drawing on healthcare professionals’ experiences, this study seeks to address this gap by providing insights into the nature of challenges in patient safety in daily ulcer management, and how unsafe situations are understood and managed to maintain and strengthen patient safety. Therefore, the aim of this study was to explore healthcare professionals’ experiences of patient safety in ulcer management in primary and secondary healthcare settings.

## 2. Materials and Methods

### 2.1. Study Design

The study employed an exploratory, qualitative design with an inductive approach, using qualitative content analysis as described by Graneheim and Lundman [40] to explore healthcare professionals’ experiences of patient safety in ulcer management. This method enabled a systematic and rigorous analysis of interview data, which facilitated an exploration of variations in participants’ experiences. The study is reported in accordance with the Consolidated Criteria for Reporting Qualitative Research (COREQ) [41].

### 2.2. Settings and Participants

The study was carried out in southern Sweden within the framework of the Wound Care project, part of the CAISR Health research environment at Halmstad University. The Wound Care project integrates advanced artificial intelligence (AI) techniques and data analytics applied to electronic health record data with qualitative interviews involving relevant stakeholders. The overall aim of the project is to investigate ulcer management pathways, uncover variations in care processes, and generate knowledge to support improvements in clinical practice. In Sweden, ulcer management primarily takes place in home healthcare or primary care. Home healthcare falls under the responsibility of the 290 municipalities, while primary care, like secondary care, falls under the responsibility of the 21 regions. If needed, specialised care units, such as vascular surgery, orthopaedics, and infectious disease clinics, can be consulted at the various stages of management [20].

For the present study, a purposive sample of healthcare professionals was recruited from primary and secondary care units in a southern region of Sweden to capture a broad range of experiences in regional ulcer management. Participants from various practice areas and professional disciplines were invited. Potential participants were identified by department heads and clinical contact persons at the participating units; in some cases, already identified professionals also suggested other colleagues with relevant experience in ulcer management. The sampling strategy aimed to ensure variation in gender, age, professional background, and years of experience. An initial invitation and written study information were sent by email, and follow-up phone calls were made to those who expressed interest in participating. All invited professionals agreed to participate, resulting in 15 participants (1 male and 14 females). Participants consisted of district nurses (*n* = 7), nurses (*n* = 4), physicians (*n* = 3), and a podiatrist in secondary care (*n* = 1) from the regional healthcare system. Ulcer management experience ranged from 0 to 45 years. Additional participant characteristics are presented in Table 1.

### 2.3. Data Collection

Interviews were conducted between January 2023 and May 2024. Experienced researchers with skills in interviewing techniques and in nursing conducted the interviews. Participants received both verbal and written information about the study and its purpose prior to the commencement of interviews and were given the opportunity to ask questions. The 15 individual interviews were conducted either face-to-face at their workplaces or via Zoom. A semi-structured interview guide was developed. The initial question was an open invitation to describe their working day, e.g., “Can you describe your job for me?” The subsequent questions were guided by the study’s aim, seeking to capture the healthcare professionals’ experiences of patient safety in ulcer management, and included prompts such as “What does patient safety mean in your work?” Follow-up questions were asked to further explore participants’ experiences of challenges and effective practices related to patient safety in their work, e.g., by asking “What are the challenges with ulcers and ulcer management?” and “How does this affect patient safety?”. All interviews were audio-recorded and transcribed verbatim. Interviews lasted between 38 and 87 min. The sample size was considered adequate in terms of information power [42], as the interviews yielded rich data with sufficient variation to address the study aim.

### 2.4. Data Analysis

To describe healthcare professionals’ experiences of patient safety in ulcer management, the transcribed interviews were analysed using qualitative content analysis as described by Graneheim and Lundman [40]. This approach allowed for the systematic identification of patterns by interpreting and abstracting the data. The analysis began with repeated readings of the transcripts to become familiar with the data and develop a comprehensive understanding of the material. Meaning units relevant to the research question were identified, condensed and then abstracted into codes, while continuously considering the context of the entire material. Both manifest and latent content were analysed, allowing exploration of the literal, explicit content and the underlying meaning of the data. Codes derived from the condensed meaning units that reflected similar meanings were grouped into preliminary categories. These categories were subsequently interpreted and discussed in relation to each other to identify underlying patterns. Based on similarities and differences among the codes and categories, an overarching theme emerged; an illustration of the analysis is provided in Table 2. During the analysis, all data was managed in Microsoft Excel (Microsoft Corp., Redmond, WA, USA). The software was used to organise the analytical steps and support systematic comparisons within and across interviews, thereby providing a transparent structure for tracking the progression from meaning units and codes to the final categories. Two researchers conducted the initial analysis, while all authors participated in iterative discussions and reflections to achieve consensus, strengthen interpretative rigour, and ensure that no significant aspects of the data were overlooked. During the process of grouping codes into categories and a theme, mind mapping was used to visualise and better understand the relationships between categories and the theme. The three categories represent different dimensions of the overarching theme and together form a comprehensive description of healthcare professionals’ experiences of patient safety in ulcer management. A visualisation of the analysis process is presented in Table 2. 

### 2.5. Qualitative Rigour

Given that qualitative research relies on nuanced subjectivity [43], multiple qualitative strategies were employed to ensure rigour. To begin with, the researchers had no prior relationship with the participants before the study commenced, which allowed them to avoid potential influence on participants’ responses and minimise social desirability bias. As researchers’ subjectivity is inherent in the research process, reflexivity on the potential benefits and limitations of the research team’s backgrounds and roles was actively integrated into the study. The research team possessed extensive clinical experience as nurses with diverse areas of expertise. This clinical pre-understanding may have sensitised the researchers to particular aspects of ulcer management and patient safety, while also carrying a risk that taken-for-granted assumptions could influence the interpretation. To manage this, emerging interpretations were discussed among all authors, and the developing categories were repeatedly checked against the interview data. All four researchers were involved in the analysis, each in a different role. The first author (MR), with specific expertise in ulcer management, conducted the primary analysis of all interviews. The last author (AG), with specific expertise in patient safety, contributed to the analysis by co-analysing data at various stages and by participating in mind mapping. Credibility was further enhanced through the involvement of the second (IL) and third (PS) authors, both senior researchers with extensive experience in qualitative methods, who reviewed and critically discussed the emerging categories. Furthermore, the two-dimensional model proposed by Graneheim et al. [44] was applied to guide the level of abstraction and interpretation, ensuring consistency across categories and the theme, while maintaining interpretations close to the empirical data. In line with recommendations by Elo et al. [45], transparency of interpretations was strengthened by illustrative quotations that encompassed a wide range of perspectives and circumstances in ulcer management, reflecting the patient safety perspective. Also, a variety of professionals and care settings were included.

### 2.6. Ethical Considerations

The study was approved by the Swedish Ethical Review Authority (no. 2022-05837-01 on the 1 December 2022, no. 2023-02581-02 on the 14 May 2023). Furthermore, the Helsinki Declaration [46] was followed to ensure the participants’ integrity and well-being. By collecting informed consent, each participant was informed about the study and their opportunity to withdraw at any time without having to provide a reason. Participation was voluntary. Information about participants and citations from interviews are presented in a way that prevents identification.

## 3. Results

### 3.1. Striving for the Patients’ Best Interests Through Individual Commitment in the Absence of Structural Support

In ulcer management, it was evident that healthcare professionals, regardless of professional affiliation, were committed to striving for the patients’ best interests, including safeguarding patient safety. Despite limited resources, both in terms of time and economic resources, ulcer healing remained a shared goal across all patient pathways. For professionals, time constraints were particularly restrictive, resulting in downgraded documentation practices, limited opportunities for continuous learning and consultation, and insufficient preventive care.

Participants demonstrated a clear awareness of systemic shortcomings and the potential implications these posed for patient safety. Although the professionals were formally part of a team, ulcer management was frequently characterised by solitary work. Healthcare professionals described feeling left alone to make critical decisions that impacted not only ulcer healing but also overall patient safety. Both individual and collective knowledge and expertise were regarded as decisive for safe ulcer management. Organisational structures, governing care provision, were likewise recognised as influential and supportive factors for professionals to ensuring the patient’s best interests.

In the absence of clear structures and guiding principles, the responsibility placed on each professional was experienced as even more pronounced. Uncertainty regarding the allocation of responsibility in ulcer management emerged as a recurring issue and was perceived as particularly challenging. The professional role extended beyond a focus on ulcer healing to encompass a broader commitment to safeguarding the patients’ best interests.

#### 3.1.1. Managing Responsibilities at the Margins of Formal Role Expectations to Promote Adequate Treatment

Healthcare professionals managed ulcer care responsibilities at the margins of their formal role. They stated that this was because of unclear distribution of responsibilities, indistinct professional boundaries, and limited access to expertise when needed, leaving questions unresolved. These organisational shortcomings were perceived as threats to patient safety, including risks of diagnostic delays, inadequate treatment plans, and omission of ulcer management, all of which could contribute to ulcer-related complications. In response, participants demonstrated a strong sense of conscientiousness, often taking personal responsibility, with responsibility for resolving issues frequently falling on individual healthcare professionals. Individual responsibility was emphasised in various ways, with an awareness that the right diagnosis and appropriate treatment largely depended on themselves. Evaluating the healing process and providing patients with accurate treatment were experienced as demanding, as was ensuring that patients were managed at the right level of care. The complexity of some patients’ situations required a clear delineation of responsibilities to determine whether the underlying disease or comorbidity should serve as the basis for referral. An accurate referral was considered a facilitator for a timely and appropriate treatment of the ulcer.

The broad, challenging, and resource-intensive nature of ulcer management placed great demands on healthcare professionals. Furthermore, some professionals reported a change over time, with a shift in responsibility leading to patients being managed to a greater extent in primary care. Sometimes, the demands on professionals approached the limit of what was reasonable. One nurse stated:

*(…) and then I have to take responsibility and send in and take samples and make a slightly more advanced assessment of whether I should send in this patient [to hospital] or not. Because we had no doctor, and then you have to take that responsibility. (…) They rely on me because I’m more experienced, and sometimes it’s at the limit.*

-Participant 1-

Given the strong emphasis on healing ulcers and the perception that responsibility for ulcer management often rested heavily on individual healthcare professionals, a sense of personal inadequacy could arise when this goal was not achieved, illustrated by one participant’s account that *’You feel inadequate when it is not healing’* (Participant 11).

In their daily work, healthcare professionals sought support from colleagues and consultants to ensure best-practice ulcer management, creating safe pathways through discussions and shared decision making. Although the importance of teamwork was widely acknowledged, barriers to effective collaboration and fragmented care pathways across healthcare settings were evident. Consequently, participants experienced patient safety in ulcer management as highly dependent on the individual engagement of healthcare professionals. A discrepancy in engagement, competence, or experience could, for example, limit the effectiveness of consultations if accurate communication regarding the ulcer was lacking, as illustrated by the following account:

*I’m a consultant for three different people from different departments who asked if we could come up and do a dressing change. And I was like, ‘no, but what does it look like?’ And they said, ‘well, I’m not really sure.’ It’s like, they just don’t have the knowledge. They can’t describe it, because ‘I don’t even know myself what I’m supposed to be looking for, so I can’t tell you what it looks like.’ Is it red? Is it swollen? Is there fibrin? Is it a blister? And they have no idea what kind of dressing to use.*

-Participant 9-

Healthcare professionals relied not only on recommendations from more experienced and competent colleagues when selecting appropriate dressings, but also, at times, on others’ assessments of the ulcers, which often necessitated in-person evaluation rather than telephone consultation. Such challenges may pose risks to patient safety, limit professional satisfaction in delivering high-quality care, and contribute to inefficient use of healthcare resources.

In summary, a strong sense of responsibility for safe ulcer management and healing extended from individual professionals to the team and, in some cases, beyond the established role expectations. An extensive, and sometimes individual, sense of responsibility risked affecting self-esteem, as healing was the ultimate indicator for good practice. Healthcare professionals tried to cover for a system with unclearly distributed and professional boundaries, all in the name of the patient’s best interests.

#### 3.1.2. Managing Ambiguous Organisational Boundaries Between Care Providers to Promote Integrated Care

Ulcer management was provided at various levels of the healthcare system, with individual professionals managing care in their own way. The responsibility between various professions was experienced as blurred, as were the responsibilities for one’s own level of the healthcare system and others’ levels of the system. It turned out that all professionals: nursing assistants, nurses, and physicians, prescribed ulcer dressings, which were sometimes considered as negative for patient safety. In a system where rotation and task-sharing were normative, not knowing who was doing what contributed to a lack of continuity, which was perceived as hindering ulcer healing and thus posing a risk to patient safety.

*There’s a patient who attends regularly for ulcer dressing, but a different professional sees them each time, and the dressing procedure varies on every occasion. No one has an overview of how or with what the ulcer is treated; decisions are made based on what feels appropriate at the time, and the ulcer never heals.*

-Participant 9-

Furthermore, low continuity was perceived as affecting the relationship between professionals and patients and the possibility of achieving a holistic overview. Also, requirements to reduce time for ulcer management were seen to reduce time for meeting the patient, at the expense of patients being unseen.

Due to time constraints, professionals’ ability to prepare for patient visits was perceived to be compromised. Additionally, the actual electronic health record systems differ across healthcare providers; critical information may not reach its intended destination, which was seen as a risk. The various providers lacked access to records from other parts of the healthcare system, risking jeopardising the ulcer management plan. Consequently, participants highlighted that individually driven examinations and treatments could expose patients to unnecessary suffering through overtreatment.

*[patients] from the municipality are from a black hole. We don’t know anything about them, beyond the referral that is sent.*

-Participant 15-

For healthcare professionals, it was obvious that more could be done to organise and structure the ulcer management, with the ultimate goal of providing safe care. Experiences from other regions or healthcare providers formed the basis for a quality assessment of the current setting. The shortcomings were perceived as being evident in the lack of clarity in patient pathways. A need for decision support and guidelines was identified to ensure the quality of both their own work and that of others. The absence of clear structures in ulcer management could mean too much was being done for patients: too many medical examinations and treatments, ultimately posing a patient safety risk, as overtreatment and unnecessary care may occur without clear benefit to the patient.

*No one really stops to consider whether the patient is actually eligible for a potential treatment, whether they are asking for treatment, or what it might lead to*.

-Participant 10-

Professionals reported that structures led to insufficient care for some ulcers. Also, there was the perception that patients with ulcers constituted an undesirable patient group. Furthermore, some ulcers, including arterial-venous ulcers, were at risk of being overlooked, as their medical affiliation might be unclear. Despite a seemingly unstructured healthcare organisation, professionals struggled to minimise risk and maximise benefits in ulcer management. The entire chain of professionals and care levels was considered vital for safe ulcer management. A lack of colleagues’ availability for workplace support was seen as a challenge to carrying out one’s professional practice. This affected the time for treatment and, consequently, patient safety, increasing the risk of unnecessary, prolonged suffering and complications. Being interrupted also posed a risk of healthcare professionals losing track of their work, as described by one participant:

*Then all the questions keep coming all the time—it’s a lot of interruptions, I think. Constantly, all the time … in the end, you don’t even remember where you are. And you have patients continuously as well.*

-Participant 7-

#### 3.1.3. Countering Overreliance on Individuals to Sustain Ulcer Management Competence

Healthcare professionals assumed personal responsibility for maintaining the expertise required to provide safe ulcer management. Limited competence was experienced as contributing to difficulties in determining accurate diagnoses and appropriate treatment, which could compromise patient safety by prolonging ulcer healing and increasing the risk of complications. In the absence of robust organisational structures to ensure collective competence, participants felt compelled to safeguard and develop their expertise independently. They acknowledged shortcomings in both their own and others’ practices, attributing them partly to insufficient preparation during basic training. Moreover, as ulcer management was perceived to be a rapidly evolving field, ongoing education was considered essential. As a result, professional development initiatives were pointed out as largely driven by individual interest and engagement, making the quality of ulcer management highly dependent on personal commitment. As one physician expressed:

*It is quite personal how much you know about ulcers. How much you get involved …*

-Participant 12-

Furthermore, participants described a lack of formalised routines to guide ulcer management, leading to increased reliance on a small number of more engaged and experienced healthcare professionals. The combination of limited formal training, dependence on a few experienced colleagues, and the expectation to maintain high standards of safety and quality of care contributed to a sense of inadequacy and exemplified how responsibility for sustaining competence had become individualised rather than structurally supported.

To promote optimal ulcer healing and maintain patient safety, participants described several compensatory strategies in everyday practice. To begin with, they had to rely on their own experience to manage ulcers. However, drawing on colleagues’ experience was important, as discussions about different ways of ulcer management were expressed as a need. Relying on colleagues’ recommendations, as well as one’s own experiences and knowledge, required a critical approach, characterised by ongoing reflection on the rationale for performing specific procedures in specific ways. Otherwise, unreflective practices could be repeatedly carried out over extended periods:

*In healthcare, I feel like you often do what someone else does. You showed me that I should always clean the ulcer in a certain way, so that’s what I keep doing—even though I don’t really know why.*

-Participant 9-

In this way, professionals aimed to minimise personal judgment. This related not only to performing tasks correctly, but also with regard to avoiding overtreatment, i.e., carrying out unnecessary investigations or procedures that could, in turn, compromise patient safety through preventable harm. Thus, a correct treatment procedure was also considered associated with insufficient competence and experience.

*It is very important to choose the correct intervention immediately to prevent the situation from escalating and becoming a major problem for the patient.*

-Participant 8-

Despite these challenges, ulcer management was described as educational, with professional development, a responsibility that sometimes extended to their leisure time. This was exemplified by a district nurse:

*So there are opportunities and then you have to take the time before or after work and things like that. But it depends a bit on you, I think, that you get to do it and I think it’s also fun, that you can. You can read up and learn new things all the time.*

-Participant 1-

Finally, assuming individual responsibility for ulcer management was ultimately motivated by the imperative to act in the patient’s best interest and to create optimal conditions for ulcer healing. However, for professionals, this reliance on individual initiative to compensate for the absence of structured competence governance also served as an informal mechanism for competence acquisition. While it ensured the continuity in ulcer care, it also perpetuated ambiguities regarding roles and responsibilities within the ulcer management process, and uncertainties about the most current evidence-based treatment regimens. Consequently, ulcer management was sustained but not always delivered in a systematic or standardised manner, which participants perceived as a potential risk to patient safety.

## 4. Discussion

With the aim of exploring healthcare professionals’ experiences of patient safety in ulcer management in primary and secondary healthcare settings, the overall findings indicated a persistent tension between the provision of safe ulcer management and the availability of organisational support and structures. The participants navigated this tension in their daily practice while striving to ensure safe ulcer management, guided by the patients’ needs. These findings suggest an organisational overreliance on individual healthcare professionals, placing them in positions characterised by, at times, an unmanageable level of responsibility. Subsequently, poor preconditions for ulcer management, such as limited formal training and lack of continuity caused uncertainty among professionals. These structural deficiencies appeared to have far-reaching consequences for safe ulcer management.

To begin with, the findings suggest that a healthcare system that places an overreliance on individual healthcare professionals to compensate for inadequate organisational conditions, thereby shifting responsibility for care quality and patient outcomes from the system to the individual. Under such circumstances, they assumed substantial responsibility for their own competency development, often extending into their personal time. Some professionals tended to take it personally when ulcers failed to heal. Such conditions have been argued to have the potential to negatively affect the work environment, which in turn, also may influence patient safety [47]. Thus, system-level interventions, such as the provision of structured competence support, may improve the work environment and, consequently, enhance patient safety, as also supported by Al-Mugheed et al. [48]. The finding that healthcare professionals have limited training in ulcer management aligns with previous research reporting perceived limitations in medical education regarding ulcers [49]. The same study reported that physicians seldom treated patients with leg ulcers and instead placed their confidence in nurses’ competence and skills, a finding that aligns with Ebbeskog et al. [49], who reported that physicians were unable to clinically develop their knowledge because they did not take an active role in ulcer management.

Furthermore, lack of diagnosis and unclear treatment strategies caused uncertainty among healthcare professionals. Additionally, deficiencies in communication within organisations, across levels of care, and in documentation further contributed to this uncertainty. These findings align with professionals’ and patients’ experiences reported in other studies [26,50]. Moreover, it has been reported that patients notice the healthcare professionals’ uncertainty, which they interpret as being caused by poor communication and continuity [36]. Patients further described the involvement of multiple staff members as contributing to fragmented care [35]. Although healthcare professionals made every effort to ensure safe ulcer management, the poor preconditions and the uncertainty they caused appear to affect not only patient safety but also patients’ perceptions of the care they receive.

Recently, national guidelines [16,18,51] for ulcer management have been developed and implemented in Sweden; however, despite the presence of guidelines and protocols, their implementation in healthcare has proven difficult [8]. Nevertheless, efforts to develop and improve ulcer management must be understood as multifaceted and complex, given the diversity of aetiologies, diagnostic pathways, and treatment strategies, as well as the involvement of multiple healthcare professionals across different levels of health care. Patient safety constitutes a collective endeavour that permeates entire organisations, from departmental leadership to frontline professionals [52]. It may also be conceptualised as a continuous, cyclical process in which professionals’ well-being and patient safety mutually influence one another [8]. Moreover, previous studies emphasise the need for initiatives to further strengthen patient safety awareness among healthcare professionals and, ultimately, reduce patient harm [53,54,55]. Acknowledging the complexity of ulcer management and strengthening system-level responsibility may be a way to relieve the burden on individual healthcare professionals, enhance patients’ perceptions of care quality, and maintain patient safety.

Moreover, the findings of this study demonstrate how healthcare professionals understand patient safety risks in ulcer management within and across healthcare settings, and how they manage these risks to support safe ulcer management and healing. In light of experienced systemic deficiencies in ulcer management pathways, competence levels, and role boundaries, it became evident that healthcare professionals routinely manage patient safety risks in everyday practice. These efforts often require substantial individual effort and do not necessarily guarantee optimal patient outcomes, particularly given the involvement of multiple actors across multiple settings. From a Safety-II perspective, professionals are indeed a resource for patient safety [38]. However, it does not seem that individual healthcare professionals’ flexibility and adaptability are enough to ensure safe ulcer healing, especially in today’s complex healthcare environment, with its rapid development, which adds to the complexity [56]. To further elucidate the types of support required, future research should focus on strengthening transitions between healthcare settings and how information, responsibility, and continuity of care are managed and coordinated across these interfaces to maintain ulcer healing and patient safety. Furthermore, policy documents and guidelines for ulcer management need to be readily available, with the necessary preconditions in place to allow healthcare professionals to put them into practice.

### 4.1. Strengths and Limitations

Aligning with the methodological preferences [40,44] in reporting trustworthiness, we use the concepts of dependability, credibility, confirmability, authenticity, and transferability. Dependability was enhanced by the use of an interview guide, and confirmability by the collaborative nature of the analysis. Credibility was enhanced by including participants with experiences of ulcer management. Furthermore, credibility and authenticity were enhanced by quotations, showing the proximity to the data and allowing others to follow the work. In this study, we included physicians, nurses, and a podiatrist from various levels of the healthcare system, each of whom contributed their experiences. As such, a strength of this study is its broad insights into the experiences of different healthcare professionals regarding patient safety in ulcer management. This mirrors the complexity of ulcer management, involving different professionals at different levels of the healthcare system. Although the researchers had no prior relationship with the participants, the focus on patient safety may have influenced what participants perceived as professionally appropriate or expected to share. Thus, experiences of uncertainty, shortcomings, or deviations from intended practice may have been more difficult to express. Given the purposive sampling strategy and the small sample, the findings may offer insights applicable to similar contexts, although their transferability should be approached with caution. Since participants were identified through department heads and clinical contact persons, it is possible that professionals with a particular interest in ulcer management, care improvement, or patient safety were more likely to be included. This should be considered when interpreting the emphasis on professional commitment and individual responsibility in the findings.

The study was conducted in a small Swedish region and included only one male participant. Despite unsuccessful efforts to recruit more men, the gender distribution was considered broadly reflective of that among healthcare professionals involved in ulcer management. Nevertheless, the limited number of male participants may have restricted the variation of gender-related experiences represented in the findings. In addition, the regional context should be considered when assessing transferability, as ulcer management pathways, responsibilities, and available resources may differ between regions and countries. However, the transferability was strengthened by the study context and the participant characteristics were described, allowing readers to assess the study’s relevance for their contexts. Patient safety, particularly in the context of ulcer management, is significant, and we believe this study contributes valuable insights to the existing body of knowledge.

### 4.2. Implications for Clinical Practice

The findings of this study suggest that improving patient safety in ulcer management requires a shift from reliance on individual professional competence towards more robust organisational support and structures. Safe care cannot depend solely on individual initiative; rather, healthcare organisations need to provide conditions that enable consistent and evidence-based practice.

Strengthening competence in ulcer management is essential, particularly in terms of diagnostic accuracy. The results indicate a need for more structured and continuous education, as well as access to clear clinical guidelines to support decision making in daily practice. In addition, systematic approaches to diagnosis should be prioritised to reduce the risk of inappropriate or delayed treatment.

The study also highlights the importance of improving communication and coordination across different levels of care. Standardised documentation practices and clearer information transfer between healthcare professionals may reduce fragmentation and enhance continuity of care.

Furthermore, organisational conditions, such as sufficient time, staffing, and leadership support are critical to enable healthcare professionals to engage in patient safety work. Creating opportunities for professionals to take an active role in safety improvement initiatives may contribute to more sustainable changes in practice.

Overall, the findings underscore the need for integrated strategies that combine education, organisational support, and improved communication processes to reduce patient safety risks and enhance the quality of ulcer management.

## 5. Conclusions

Healthcare professionals involved in ulcer management show a clear awareness of patient safety risks and actively strive to mitigate potential harm through their awareness, knowledge, and commitment to patients’ well-being. However, the findings indicate that ensuring safe care often depends on individual efforts, placing substantial demands on healthcare professionals. Organisational constraints, such as limited time and insufficient coordination across different levels of care, were perceived as barriers that may compromise patient safety. These findings suggest a need for stronger structural support to complement individual competence, as patient safety in ulcer management should not depend solely on individual healthcare professionals. Further research should explore how organisational structures and interprofessional collaboration can be strengthened to support patient safety in ulcer management, including patients’ experiences of safety and comparisons of how ulcer management is organised across different healthcare systems and professional contexts. Studies of patient flows, for example, through patient records, could also provide insights into how organisational conditions relate to patient safety risks and outcomes in ulcer management.

## Figures and Tables

**Table 1:** Participant characteristics.

**Gender** (*n*)	
Man	1
Woman	14
**Age** (years, mean)	31–64 (52.6)
**Profession** (*n*)	
Nurse	4
District nurse	7
Physician	3
Podiatrist	1
**Occupation** (*n*)	
Primary care	8
Secondary care (inpatient)	2
Secondary care (outpatient)	4
Secondary care (outpatient and inpatient)	1
**Experience of ulcer management** (years, mean)	0–45 (15.87)
**Extent of ulcer management** (*n*)	
Daily	5
Weekly	7
Occasionally	1
Not currently	2
**Educational background in ulcer management** (*n*)	
University-level education only	9
University-level education + workplace in-house training	3
University-level education + university continuing education	3

**Table 2:** Analysis process with the overarching theme: Striving for the patients’ best interests through individual commitment in the absence of structural support.

Citations	Condensed Meaning Units	Codes	Categories
It feels like ulcer patients are always caught in the middle. Primary care keeps them for quite a long time, and then, when they finally need to refer them, they don’t really know where to send them. And it’s a very vague boundary, who should go to orthopaedics, who should go to the surgeon, who should go to dermatology? [Participant 9]	Primary care tends to manage these patients for quite some time, but when referral becomes necessary, there is often uncertainty about where to send them. As a result, patients often get caught in the middle.	To not clearly fit anywhere and get caught in between	Managing responsibilities at the margins of formal role expectations to promote adequate treatment
We have a vascular consultation phone line, and it’s mainly used, for example, by diabetic patients coming in for regular dressings. There are patients we absolutely must not miss, and in those cases … You can deviate from the guidelines; it works perfectly well to get in touch directly. [Participant 1]	There are patients we absolutely must not miss, and in those cases, you can deviate from the guidelines	Guideline deviation to safeguard against missed patients	
We often end up doing loads of other things that the patient probably doesn’t actually need [in ulcer management]. We do lots of tests, take loads of samples, start treatments that aren’t really asked for. And often it just leads to a lot of complications and suffering. [Participant 1]	A lot gets done without knowing what the patient actually wants, which can cause suffering	Individually driven ulcer management risks causing suffering	Managing ambiguous organisational boundaries between care providers to promote integrated care
You do the best you can, but there isn’t always enough time. And I think it’s kind of lacking when it comes to getting input and updates, that’s hard to come by. You have to actively look for it yourself, and … well, you just don’t have the time. It ends up being those moments when you think, ‘this isn’t working, what else can I try?’ [Participant 7]	It’s hard to keep up to date with ulcer care because there just isn’t enough time. Everyone must take responsibility for staying up to date.	Even though time is limited, engaged professionals keep up to date.	Countering overreliance on individuals to sustain ulcer management competence
No, not really [when asked about formal training], except self-taught or learning from colleagues. And then you’ve been to different lectures, ulcer care symposiums, that kind of thing. And the doctors have taught us how to do things, too. [Participant 5]	Making up for the lack of formal training with whatever resources are available.	Coping with not having enough training	

## Data Availability

The data presented in this study are available on request from the corresponding author due to privacy and ethical restrictions.
